# Light-addressable Electrodes for Dynamic and Flexible Addressing of Biological Systems and Electrochemical Reactions

**DOI:** 10.3390/s20061680

**Published:** 2020-03-17

**Authors:** Rene Welden, Michael J. Schöning, Patrick H. Wagner, Torsten Wagner

**Affiliations:** 1 Institute of Nano- and Biotechnologies (INB), Aachen University of Applied Sciences, Heinrich-Mußmann-Str. 1, 52428 Jülich, Germany; welden@fh-aachen.de (R.W.); schoening@fh-aachen.de (M.J.S.); 2 Laboratory for Soft Matter and Biophysics, KU Leuven, Celestijnenlaan 200D, 3001 Leuven, Belgium; 3 Institute of Complex Systems (ICS-8), Research Center Jülich GmbH, 52428 Jülich, Germany

**Keywords:** light-addressable electrode, light-addressable cell stimulation and photoelectrochemistry, photoelectrochemical deposition

## Abstract

In this review article, we are going to present an overview on possible applications of light-addressable electrodes (LAE) as actuator/manipulation devices besides classical electrode structures. For LAEs, the electrode material consists of a semiconductor. Illumination with a light source with the appropiate wavelength leads to the generation of electron-hole pairs which can be utilized for further photoelectrochemical reaction. Due to recent progress in light-projection technologies, highly dynamic and flexible illumination patterns can be generated, opening new possibilities for light-addressable electrodes. A short introduction on semiconductor–electrolyte interfaces with light stimulation is given together with electrode-design approaches. Towards applications, the stimulation of cells with different electrode materials and fabrication designs is explained, followed by analyte-manipulation strategies and spatially resolved photoelectrochemical deposition of different material types.

## 1. Introduction

Charge transfer is the main task of working electrodes in electrochemistry, while they are in steady interaction with an electrolyte [1,2,3,4]. The overall application field of biosensors [5,6,7] extends from enzymatic biosensors [8,9,10] and impedimetric DNA sensors [11] to detection of action potentials of neurons with microelectrode arrays [12]. Depending on the application, electrode sizes range from macro- and micro- down to nanoelectrodes with adjustable geometries [13,14,15]. Regarding fabrication methods, such as thick- and thin-film technologies, several process steps are necessary. For example, in thin-film technologies, electrode design has to pass elaborated steps from photolitography to material deposition. As materials, noble metals such as platinum, gold, or silver are the most common electrode materials; however, it is also possible to use carbon [16,17], graphene [18,19,20], metal oxides [21,22,23], or conductive polymers [24,25,26]. Despite of their wide application field and highly developed technology standards, those electrodes are limited in their flexbility as they are usually tailored to a specific task, and due to that, they need often highly sophisticated fabrication- and read-out procedures. If a specific electrode design has to be integrated, for instance, in a lab-on-a-chip system, each single development step might require changes due to the electrode design or location. This causes a time- and resource-consuming redesign of the electrodes and the lab-on-a-chip system with an adjustment of fabrication steps. In addition, the modification of other components can have an influence on the electrode structure itself, such as the wiring of the connection.

Instead of using these classical electrode materials, semiconductors obtained attention as alternative materials for electrochemistry from first studies by Gerischer [27] and for photoinduced water splitting by Fujishima and Honda [28]. When a semiconductor is brought into contact with an electrolyte, charge carriers will exchange between the semiconductor and electrolyte until an equilibrium is reached. This is followed by a band alignment (between the valence band $E_{g}$ and the conduction band $E_{c}$) in the semiconductor, where a space-charge layer is formed at the interface. When light with a suitable wavelength (photon energy > band gap energy) is absorbed by the semiconductor, electron-hole pairs can be generated. Depending on the doping, the minority charge carriers, holes (h^+^) for n-type semiconductors and electrons (e^−^) for p-type materials, will mainly contribute to the charge transfer at the semiconductor–electrolyte interface, where redox reactions can occur:

(1) $$Red+h^{+}\to Ox$$

(2) $$Ox+e^{-}\to Red$$

**Figure 1:**
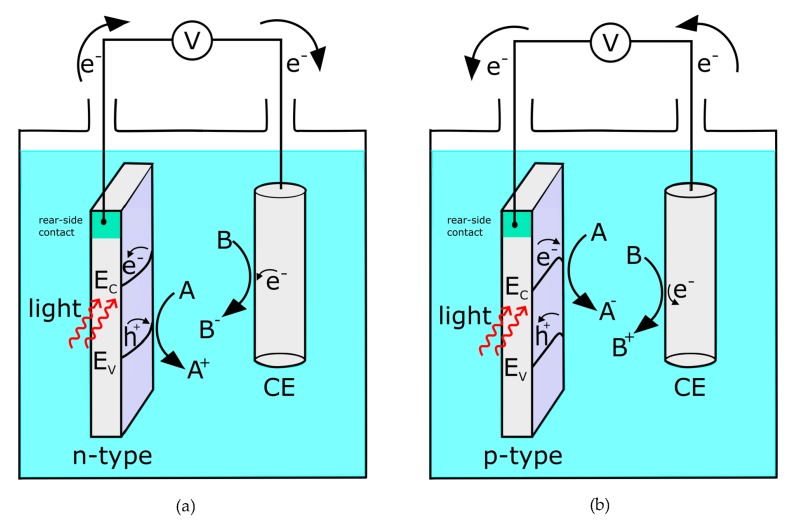
(**a**) n-type semiconductor as a photoanode in contact with a counter electrode (CE): At the photoanode, substance A is oxidized by the photogenerated hole, while the electron is moved to the counter electrode where it can reduce substance B. (**b**) p-type semiconductor as a photocathode in contact with a counter electrode: At the photocathode, substance A is reduced by the photogenerated electron, while the hole is moved to the counter electrode where it can oxidize substance B.

In Equation (1), a reducing agent (Red) is oxidized by releasing electrons, while in Equation (2), an oxidizing agent (Ox) is reduced by gaining electrons. For example, for a n-type semiconductor, by applying an anodic potential, electrons (majority charge carriers) will move to the bulk while the holes (minority charge carriers) can perform oxidation reactions at the surface. Therefore, such a structure is called a photoanode. Opposite reactions take place at a p-type semiconductor, a photocathode. A detailed explanation of the related semiconductor physics is given in References [29,30]. The schematic working principles of photoanodes and -cathodes are shown in Figure 1.

**Figure 2:**
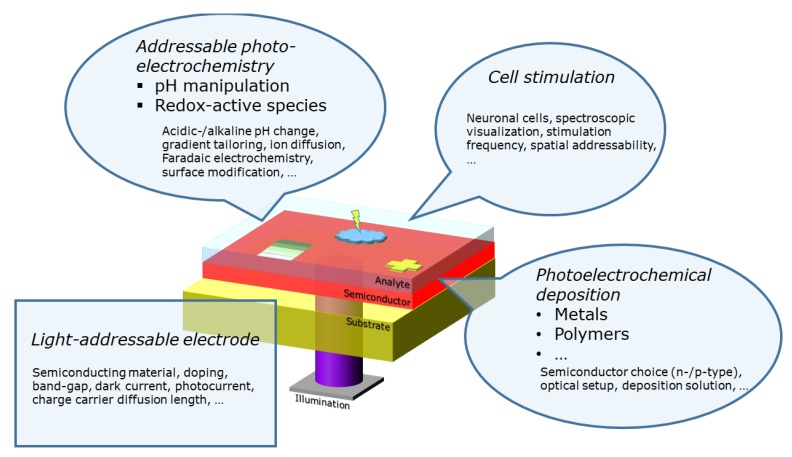
Overview of the presented topics in this review article: In contrast to well-known electrodes made of noble metals, light-addressable electrodes with a semiconductor electrode material can be addressed with a light source. Addressable photoelectrochemistry, cell stimulation, and photoelectrochemical deposition are introduced as possible applications.

A more extensive theoretical introduction about light-addressable electrochemistry is given by Vogel et al. [31]. Well-known examples are photoelectrochemical cells [32,33] and dye-sensitized solar cells [34], to which a tremendous amount of reseach is dedicated. Nevertheless, also other fields were established based on photoelectrochemistry: photoelectrochemical sensors [35,36,37], for various applications, including DNA (deoxyribonucleic acid) [38], immuno [39], enzymatic [40], and heavy metal sensing [41] are known in the literature. Mainly to trigger detection, those chips are fully illuminated with a single light source and the possibility of addressing multiple analytes or areas of the sensor is disregarded. The potential of obtaining spatially resolved information on the analyte concentration was demonstrated with light-addressable potentiometric sensors [42,43,44]. In contrast to photoelectrochemical sensors, an insulating layer is deposited on top of the semiconductor to prevent direct charge transfer at the semiconductor–electrolyte interface. Applying a d.c. (direct current) potential, a space-charge region will be formed at the semiconductor–insulator interface. The width of this space-charge layer will change according to the ion concentration at the sensor surface. Using an intensity-modulated illumination, electron-hole pairs are continuously generated and separated in the space-charge region. Hence, the resulting alternating photocurrent is proportial to the width of the space-charge region and therefore to the ion concentration. By scanning the sensor with an appropiate optical system, spatially resolved sensor images of the analyte concentration, so-called chemical images, can be obtained. In chemical and biological systems, not only the detection of analytes or cells is of importance. An additional changing of the local environment to trigger a chemical reaction or manipulating a cell can also be significant. Especially, the localized dynamic triggering with a light source in, e.g., a lab-on-a-chip system, can be beneficial in comparison to conventional electrodes, where a sophisticated design and layout is necessary. One well-known technique which utilizes this idea is optoelectronic tweezers [45,46,47]. They are based on dielectrophoretic techniques by applying a.c. (alternating current) voltages between the photoconductive surface and a counter electrode, which results in nonuniform electric fields at the illuminated areas. Particles in that area might interact due to their dipoles with the electric field as dielectrophoretic forces acting on these particles. Hereby, a controlled movement of molecules or even biological cells is possible. Our review will give a closer look at three additional light-addressable electrode applications: (i) stimulation of cells,(ii) addressable photoelectrochemistry and, (iii) photoelectrochemical deposition (Figure 2).

**Figure 3:**
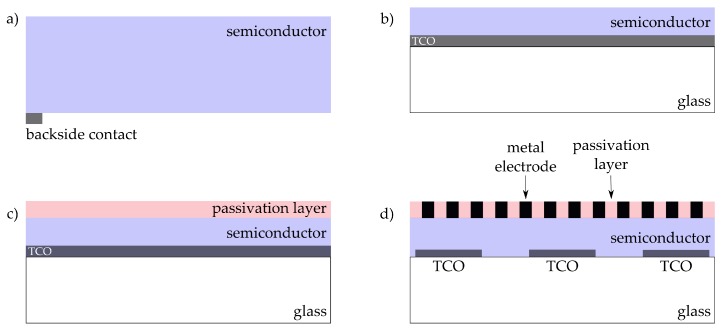
Different design possibilities for light-addressable electrodes: (**a**) Pure semiconductor electrically contacted, (**b**) semiconductor film deposited on a transparent conductive oxide (TCO) glass substrate for electrical connection, (**c**) deposited passivation layer for protecting the semiconductor film against environmental conditions, and (**d**) integrated metal electrodes in the passivation layer (for charge exchange) deposited on the semiconductor film.

For a successfull implementation in an experimental setup, the photoelectrode design and the optical system are the major contributors. In literature, different keywords are used for the electrode description, e.g., “photoelectrochemical”, “light-induced”, “light-directed”, “optically directed”, “photo-assisted”, etc. For simplicity, the actual working electrode will be called light-addressable electrode (LAE) in this review. The design of those electrodes—see Figure 3—relies mainly on the selected semiconductor. Depending on the application, the conduction and valence band energies have to fit to the reduction and oxidation potentials of the analyte, whereby the band gap also defines the possible excitation wavelength. A detailed calculation of common band energies is given in Reference [48]. Furthermore, a sufficient dark-to-photocurrent ratio and the charge-carrier diffusion length of the semiconductor are essential to trigger a reaction only at the area of illumination. A semiconductor can be directly electrically connected (Figure 3a) or deposited as a thin film on a substrate material (Figure 3b). Often, transparent conductive oxide (TCO) glasses such as indium tin oxide (ITO) [49], fluorine-doped tin oxide (FTO) [50], or aluminium-doped zinc oxide (AZO) [51] are used as substrate materials as they allow rear-side illumination and easy electrical connection. Since LAEs are mainly used with liquid environments, the semiconductor material should be stable under the prevailing conditions. Especially the stability in alkaline or acid solutions has to be taken into account as the degradation/corrosion of the material is possible [52]. A discussion about the stability for a wide range of semiconducting materials which can be used as LAEs can be found in Reference [53,54]. Nevertheless to improve the stability, e.g., for silicon or amorphous silicon, a passivation layer can be applied (Figure 3c). The passivation layer has then to be thin enough for charge tunneling; anisotropically conductive or redox groups are required. It is also possible to integrate single noble-metal electrodes electrodes into the passivation layer to have a charge path to the analyte (Figure 3d).

In addition to the electrode design, a proper illumination source is required for flexible addressing of the LAE. In the beginning, single-focus laser spots were used for spatial illumination by mounting them onto motorized stages [55], or dimensional excitation was done by photomasks [56]. A more dynamic and flexible addressing can be achieved with Digital Micromirror Device (DMD) [57] or Ferroelectric Liquid Crystal on Silicon (FLCoS) [58] technologies. By individual switching of single pixels, both technologies (DMD and FLCoS) combine the flexibility of a scanning laser and the extensive pattern illumination of a photomask. Nevertheless, a sufficient optical focussing has to be provided to project small-scale patterns onto the electrode. In the next section, a more detailed look on the electrode design, integration of optical systems, and example applications for the stimulation of cells by the LAE will be given.

## 2. Stimulation of Cells

Multielectrode arrays (MEA) are well established for manipulating cells and for recording their electrical potentials [12]. From first MEAs with 30 microelectrodes to record potentials from chicken heart cells [59] to arrays based on CMOS (complementary metal-oxide-semiconductor) technology, which have bidirectional functionality for recording and stimulation with 26,400 electrodes [60], considerable research efforts were dedicated to those electrodes. The incentive for developing new kinds of electrodes are the existing challenges. Wiring of single, fixed-positioned metal electrodes led to a limited density of electrodes with the consequence that cells lying between electrodes, or barely covering them, could only be monitored with a low signal-to-noise ratio. The high electrode density and spatial resolution, together with the good signal-to-noise ratio of nowadays CMOS technology, goes, however, along with very sophisticated fabrication and signal processing. The high costs for these MEA chips, fabricated in small numbers, make them not attractive for many potential applications. Especially, cell-culture-based applications, which need steril environments, prefer disposable systems.

Therefore, as an alternative to these technologies, light-addressable electrodes were introduced for cell stimulation by Colicos et al. [61]. In this work, photoconductive stimulation of cultured neurons was perfomed to image the green fluorescent protein (GFP) actin at individual synapses to show synaptic plasticity. A p-doped silicon wafer was used as a photoconductive material with a platinum counter electrode. For neuronal stimulation, square voltage pulses (4 V) were applied between both electrodes, while the desired photoconductive pathway was continously illuminated with a small spot (∅ = 80 μm) from an upright microscope. This experimental setup was also applied to stimulate rat hippocampal neurons in vitro. The direction of growth cones of axons was affected by triggered astrocyte Ca^2+^ waves [62]. Furthermore, different postsynaptic proteins of rat hippocampal neurons were studied at various photoconductive stimulation frequencies [63].

**Figure 4:**
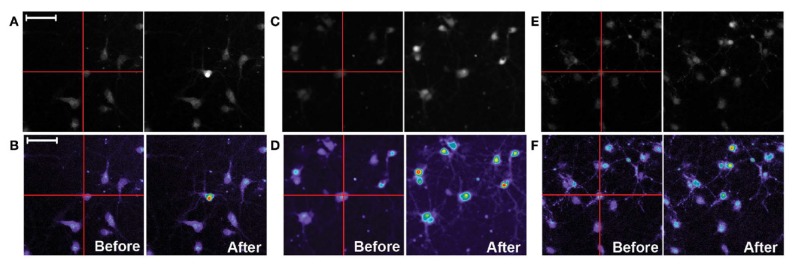
Fluo-4 calcium fluorescence images of rat primary cortical cells stimulated from the top-side by light-addressable electrodes with different semiconductor materials: (**A,B**) polished silicon, (**C,D**) porous oxidized silicon, and (**E,F**) porous carbonized silicon. On the left side of each group of four images, the grayscale image (top) and related heatmap image (bottom) before stimulation with a laser source is shown, while on the right side, the images of the stimulated neurons are depicted. The scale bar indicates 50 μm. Adapted from Reference [64] under the CC BY licence.

The technique was further improved by Campbell et al. [64]: A measurement chamber allowing for integration within an inverted confocal microscope was applied and the influence of different silicon substrates (polished p-doped silicon, porous oxidized p-doped silicon, and porous carbonized p-doped silicon) was evaluated. Rat primary cortical neurons were cultivated on the different substrates and stimulated; the Ca^2+^ response due to the stimulation was visualized with a Fluo-4 fluorescence dye. For all three substrates, the fluorescence signal of the stimulated neurons followed the stimulation frequency from the photoconductive material when pulsing the applied voltage under a constant illumination. Figure 4 shows the fluorescence signal before and after selective stimulation of neuronal cells. A single cell (marked with the crosshair) was stimulated by a laser spot, and the fluorescence increase of the adjacent cells was observed. For polished silicon, only 20% of the cells in an area of 25 μm away responded. For oxidized silicon, around 30% in a distance of 75 μm and, for carbonized silicon, 20% of the cells in a range of 200 μm away from the stimulated area are activated. This work demonstrates that, depending on the application (single-cell stimulation or stimulation of a group of neurons), different photocondutive materials can be chosen.

Silicon as an photoconductive material, which tends to oxidize without special treatment under environmental conditions, can be replaced by hydrogenated amorphous silicon (a-Si:H) to improve the spatial resolution. It is possible to deposit amorphous silicon as thin layers on a substrate material, which improves the spatial resolution due to limited lateral diffusion of the electron-hole pairs [65]. One challenge of a-Si:H is its unstable behavior in liquid environments, meaning that a passivation layer is required. The first electrodes with a-Si:H photoconductive material were introduced by Bucher et al. [66,67,68]. Hydrogenated amorphous silicon was deposited on a patterned (60 rows, 20 μm wide, 10 μm gap) indium tin-oxide (ITO) glass substrate, which was used for electrical connection. To protect the photoconductive film, Ti–Au or TiN electrodes were patterned on each ITO lead. As a final step, a SiO_*x*_:C insulating layer was fabricated between the single electrodes for surface passivation. Without electrolyte, the Ti–Au electrodes showed a d.c. ohmic behavior in the range between −0.2 V to 0.2 V with a dark (without illumination) to bright (with illumination) resistance ratio of 10^7^ and an a.c. impedance ratio (dark/bright) of $5\cdot 10^{6}$ for the TiN electrode. In both cases, illumination of the neighbouring electrode resulted in a decrease of the dark resistance (d.c.) resp. impedance (a.c.) of the electrode under study due to light scattering. In electrolyte (physiological buffered solution), the a.c. impedance ratio (dark/bright) of the TiN electrode decreased to 30–60. Nevertheless, it was possible to record signals from cardiac myocytes cultivated on the electrodes’ surface when they were illuminated.

The deposition and patterning of single electrodes in the previous design requires a sophisticated fabrication method with sputtering, Ar-plasma-etching, plasma-enhanced chemical vapour deposition (PECVD), and CF_4_-O_2_-plasma-etching. The electrode design was simplified by Suzurikawa et al. by using an anisotropic passivation layer [69,70]. For electrical connection, a glass substrate with transparent SnO_2_ was used with a thin a-Si:H (150 nm) photoconductor on top. To prevent dissolving, low-conductive zinc-antimonate (ZnOSb_2_O_5_)-dispersed epoxy was deposited on the a-Si:H by spin-coating and subsequent baking steps. The low water absorption of the epoxy resin prevented the culture medium from dissolving the underlying a-Si:H layer. The sheet resistance of the film was 10 MΩ/sq., and it was stable for more than two weeks in cell-culture medium. Without illumination, the charge density increased exponentially with increasing bias voltages (negative monophasic 1 ms pulses, 0–9 V), while the charge density showed a linear increase for increasing voltages under illumination. At 3 V, the best dark-to-bright charge ratio was achieved with a factor of 60. Neurons from Wistar rat embryos were plated and cultivated on the electrodes, and the stimulation was evaluated by fluorescence with Fluo-4 calcium images. For stimulation, negative voltage pulses (3 V, 1 ms) were applied between the SnO_2_ layer and a counter electrode at a constant illumination (∅ = 200 μm, 800 mW/cm^2^). An increase in the fluorescence signal around the illuminated area confirmed the successful stimulation of the neurons. In further studies, a light intensity between 400 and 800 mW/cm^2^ was found to be sufficient for stimulation and it was possible to control stimulation pulses with a frequency of at least 500 Hz. To activate neuronal cells, a minimum charge density of 10–20 μC/cm^2^ was estimated, and for single-cell stimulation, the spatial resolution was found to be 10 μm [71]. To improve the electrode performance, the dependence of the photoconductor thickness (d = 50 nm, d = 150 nm and d = 1000 nm) and two passivation layers, zinc-antimonate-dispersed epoxy (ZADE, d = 2 μm, 10 MΩ/sq.) and poly(3,4-ethylenenedioxythiophene) (PEDT, d = 0.5 μm, 4 kΩ/sq.), was analyzed. The results indicate that the performance can be further improved by a thicker photoconductive layer and by using a passivation layer which has almost the same resistivity as the photoconductive layer under illumination [72].

**Figure 5:**
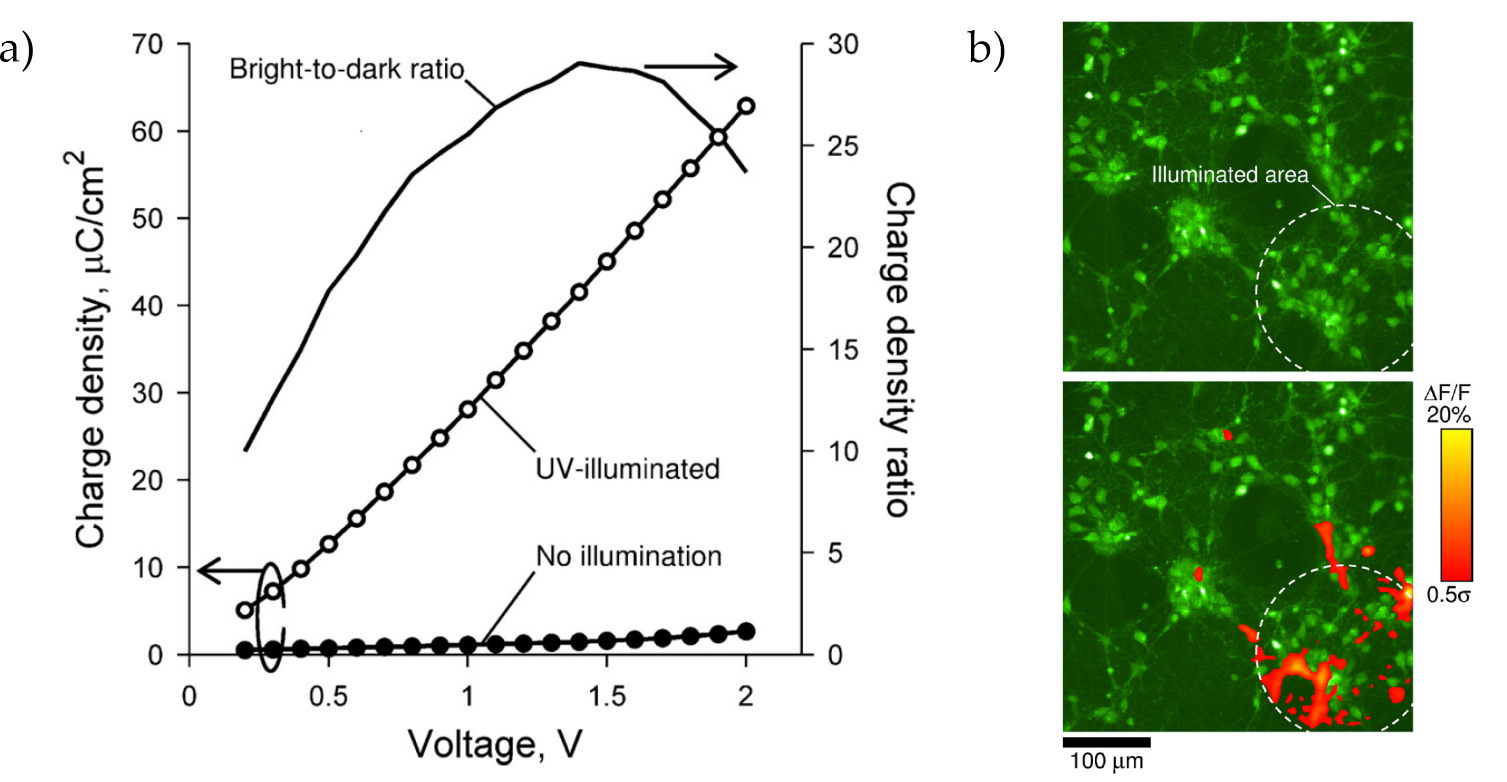
(**a**) Example of the charge density for a porous titanium dioxide light-addressable electrode with different, applied voltages under and without illumination. (**b**) Ca^2+^ fluorescence image of rear-side illuminated spatial stimulation of Wistar rat embryo neurons cultivated on titanium dioxide: Neurons show fluorescence changes at the illuminated areas. Reprinted from Reference [73] Copyright (2020), with permission from Elsevier.

In another approach, Suzurikawa et al. replaced the photoconductive a-Si:H passivation sandwich structure by titanium dioxide [73]. TiO_2_ anatase nanoparticles were deposited on a conductive fluorine-doped tin oxide (FTO) glass by spin-coating and subsequent sintering at different temperatures (350 °C, 500 °C and 500 °C with TiCl_2_ treatment). For photoelectrical characterization, 1 ms negative voltage pulses were applied from 0.2 to 2 V against a Pt electrode. Without illumination, charge densities were lower than 3 μC/cm^2^, which is below the suggested threshold of 10–20 μC/cm^2^ for neuron stimulation. The best dark-to-bright ratio was achieved at 1.4 V with a factor of 29 (Figure 5a). Due to the mesoporous structure of the TiO_2_ film and enhanced surface hydrophilicity by long-term illumination, the charge density without illumination was increased as the electrolyte solution penetrated through the film and stayed eventually in direct contact with the FTO. Furthermore, the charge density during illumination was increased with slow rising times, whereas by turning off the illumination, slow charge density decays can be observed due to the low recombination rates of photogenerated charge carriers. Nevertheless, the rising times and amplitudes of the charge density with illumination can be improved with higher sintering temperatures and TiCl_2_ treatment. For stimulation, neurons from Wistar rat embryos were cultivated and stimulation was evaluated by Fluo-4 calcium images. Applying a 1 ms, −1.5 V voltage pulse leads to a charge density of 44 μC/cm^2^ for illumination and 10 μC/cm^2^ in dark regions. This was suitable for spatial resolved neuronal stimulation as seen in the increased fluorescence signals (Figure 5b).

In summary, the possibility to spatially address and trigger single cells by using light-addressable electrodes was shown in this section. For choosing the best suited material, the stability in aqueous environments, the dark-to-bright current ratio, and the spatial resolution have to be considered. If the photoconductive material is not stable in aqueous conditions, a passivation layer can be added. This in turn can influence the respective dark and photocurrents and can therefore improve or impair the dark-to-bright current ratio, which is necessary to stimulate the cell. The choice of materials can also have an influence on the spatial resolution, since the diffusion length of the generated electron-hole pairs contribute to the addressed area and therefore directly influences the addressability.

## 3. Addressable Photoelectrochemistry

The development of lab-on-a-chip microfluidics requires besides sensing [74,75] and flow-control elements [76] also active manipulation structures. One possible parameter to control inside those microfluidic systems is the pH value. Besides active measurements [77,78], different techniques, e.g., a.c. Faradaic reactions [79], field-enhanced water dissociation in microscale bipolar membranes [80,81], or electrolysis at electrodes inside the channel [82,83] have been developed for active manipulation of the pH value inside those microstructures. For water electrolysis, where not only gas, but also protons and hydroxide ions are generated, the anodic (Equation (3)) and cathodic (Equation (4)) reactions can be described as follows:

(3) $$2\;H_{2}O\to O_{2}+4\;H^{+}+4\;e^{-}$$

(4) $$4\;H_{2}O+4\;e^{-}\to 2\;H_{2}+4\;OH^{-}$$

Instead of using noble metal electrodes for electrolysis, which have to be arranged in predefined locations, a more versatile method to control different pH values inside the analyzed system can be the use of photoanodes or photocathodes made of semiconductor materials. Especially, in combination with a sophisticated illumination system, a fast and spatially resolved pH changing system can be introduced. This principle was used by Hafeman et al. in his work about photoelectophoretic localization and transport (PELT) [84]. This technique enables the generation of electrical force-field traps for charged molecules by illumination on photoconductive electrodes. In combination with this, a three-dimensional positioning of amphoteric molecules by a surface pH gradient can be established. As photoanode materials, titanium dioxide (d = 6 μm) on transparent conductive ITO glass and germanium (d = 360 μm) photoanodes were used. Due to photoelectrolysis, the pH gradient can be controlled by illumination intensity, duration, and position. As reported, amphoteric molecules (negatively charged Bovine serum albumin) will be first attracted by the illuminated isoelectric zone. After charge exchange and more acidic conditions, they will turn the direction away from the surface. Therefore, it is possible to influence the vertical position of the molecule related to the surface in space by the pH gradient.

**Figure 6:**
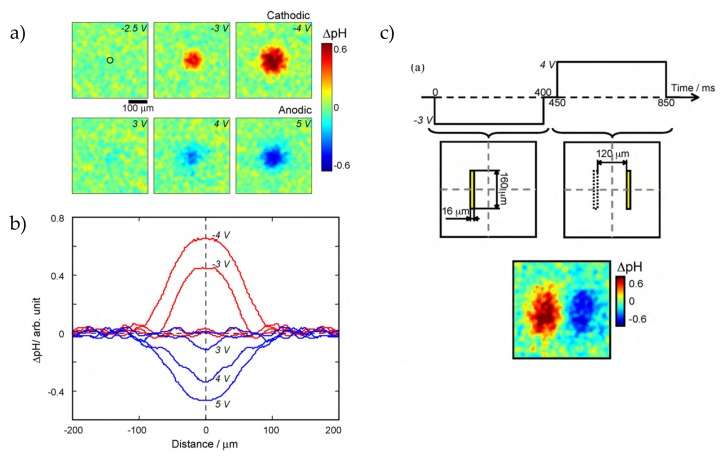
(**a**) Fluorescence pH images for a-Si:H with a tin antimonate (ZnO/Sb_2_O_5_)-dispersed epoxy passivation layer for different applied potentials with a constant rear-side illumination: For negative applied potentials, the pH changes to more alkaline value, while for positive applied potentials, the electrolyte becomes more acidic at the illuminated areas. (**b**) pH gradients for different potentials: With higher positive or negative potentials, the gradient gets wider with an absolute higher pH change. (**c**) Customized pH gradient by first applying a cathodic potential and illuminating the first area for 400 ms, where the pH gets more alkaline, followed by switching to anodic potentials for additional 400 ms together with the illumination of the seconds area. Reprinted from Reference [57] Copyright (2020), with permission from Elsevier.

Suzurikawa et al. had a more detailed look at pH gradient generation on photoelectrodes [57]. In short, a-Si:H was deposited on a fluorine-doped SnO_2_ (FTO) glass with a tin antimonate (ZnO/Sb_2_O_5_)-dispersed epoxy passivation layer. Changes of the pH value were imaged by 2’,7’-bis-(2-carboxyethyl)-5-(and-6)-carboxyfluorescein (BCECF). Voltage pulses of 400 ms from −4 V to 5 V between the photoelectrode and a counter electrode were applied. No pH changes were found for voltages between −2.5 V and 2.5 V. At −4 V, the pH change (ΔpH) was 0.65 and −0.45 at 5 V (Figure 6a). Due to the PBS (phosphate buffered saline) buffer, chlorine evolution dominates the anode reaction where protons are generated and the pH value becomes more acidic whereas hydroxide ions are directly generated at the cathode. A DMD projector was integrated in the measurement setup to generate flexible illumination patterns. Beside the pH value, also the width of the pH gradient changes with the applied voltage (Figure 6b). It was found that the pH change increases with the illumination width under same pulse durations and that the full width at half maximum (FWHM) of the ΔpH gradient profile correlates with the ΔpH peak value. By applying cathodic and anodic pulses with simultaneously defined illumination patterns, it was also possible to tune the pH gradient profiles due to neutralization reactions of hydroxide ions and protons. Furthermore, by sequentially illuminating two different areas for 400 ms, a wide-range pH gradient could be achieved when applying in the first area a cathodic potential (−3 V, +ΔpH) and in the second area an anodic potential (4 V, −ΔpH) (Figure 6c).

This experimental setup with a DMD projector as the illumination source was also used with a glass/ITO/n-doped a-Si:H/undoped a-Si:H or glass/ITO/titanium dioxide phthalocyanine (TiOPc) structure to manipulate the pH values by photoelectrolysis for spatial deposition of calcium alginate [85,86] and chitosan [87,88]. For the a-Si:H structure, the pH change was monitored by a pH-sensitive fluoerescence dye (BCECF) diluted in DI water and an applied current density of 4 Am^−2^. After 30 s of illumination, sharp fluoerescence images can be observed with a pH value higher than pH 6.3 while the non-illuminated areas remained at pH 5.5. After 120 s, the sharp fluoerescence image got blurred due to diffusion [87].

**Figure 7:**
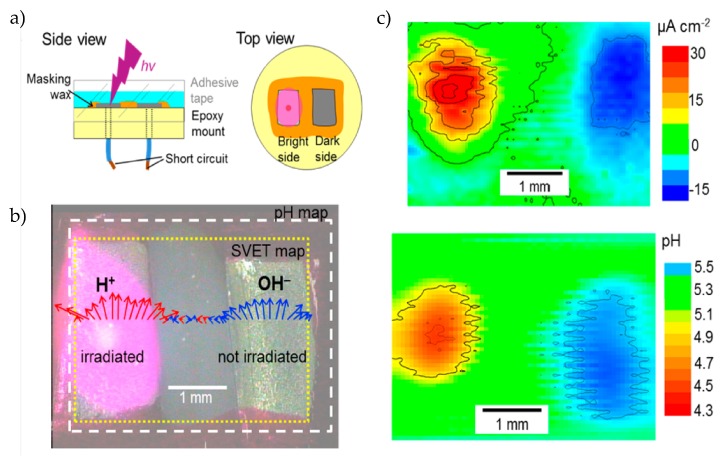
(**a**) Two separated titanium dioxide electrodes (n-type semiconductor) in one measurement chamber: Both electrodes are electrically shortened, while one electrode is illuminated from the top-side. (**b**) Due to the n-type behaviour, protons (H^+^ ions) will be generated at the irradiated electrode, while the opposite reaction (OH^−^ ions) will occur at the non-illuminated electrode. The arrows indicate the current density for the anodic (red) and cathodic (blue) reactions. (**c**) Current and pH maps of both electrode regions: Positive photocurrent occurs only at the illuminated area, while at the non-irradiated electrode, a negative current is present over the complete surface. The pH changes correlate with the current map. Reprinted (adapted) with permission from Maltaneva et al. [91] Copyright (2020) American Chemical Society.

In other works, based on titanium dioxide [89,90,91], a single electrode setup was used without applied potential to photocatalytically change the pH value at illuminated regions. Water oxidation and reduction is catalyzed by photogenerated charge carriers. Due to the n-type behaviour of TiO_2_, protons are generated by oxidation while electrons are transported to non-illuminated areas, where they recombine or get trapped by scavanging agents. To quantify the pH change, measurements by scanning ion-selective electrode technique (SIET) were performed to locally scan the proton generation on the electrode surface. With a nanotubular TiO_2_ film, under illumination in Na_2_SO_4_, a pH change from pH 5.5 to pH 3.6 due to proton generation have been observed. The relaxation time of the pH value is around 40 min, while the photocurrent relaxes in 1–3 min. In the z-direction, the pH change is linearly in the range of 0–350 μm, while there is a nonlinear change from 350 to 1000 μm above the electrode surface. As depicted in Figure 7a,b, two separated electrodes (short circuited) are arranged in one chamber whereby only one electrode is illuminated. At the illuminated electrode, H^+^ ions will be generated and the electrolyte gets more acidic, while at the non-illuminated electrode, the opposite reaction occurs (OH^−^ ion generation) and the pH is more alkaline. The arrows indicate the current density measured with the scanning vibrating electrode technique (SVET) (Figure 7b). The pH and current distribution heatmaps at the electrode areas measured by SIET and SVET are shown in Figure 7c. In the pH heatmap, the pH change at the illuminated electrode is concentrated in the area of illumination, while at the non-illuminated electrode, the pH change is distributed over the whole electrode, which is in coincidence with the current density heatmap [91]. In related work, a pH change from pH 6 to pH 5.6, pH 4.5, and pH 4 after 5 s, 1 min, and 3 min was observed [90]. This pH change can be used to modify the structural properties of pH-sensitive layer-by-layer (LbL) assembled polymer films. For the LbL structure, a positively charged poly(acrylic acid) (PAA) and a negatively charged pH-sensitive ABC triblock terpolymer, which self-assembles to core-shell-corona micelles, were deposited on titanium dioxide. Due to the pH-sensitive poly(methacrylic acid) shell, locally induced pH changes led to an increase of the thickness and a softer LbL structure.

Another principle for locally induced photoelectrochemistry by light was established by Choudhury et al. who termed the technique “light-activated electrochemistry” [92]. It is based on functionalization of redox-active species on oxide-free silicon surfaces to perform light-addressed Faradaic electrochemistry on the electrode surface [93,94]. For the electrode preparation, monolayers of 1,8-nonadiyne were assembled on pretreated n- and p-doped Si(100) wafers.

**Figure 8:**
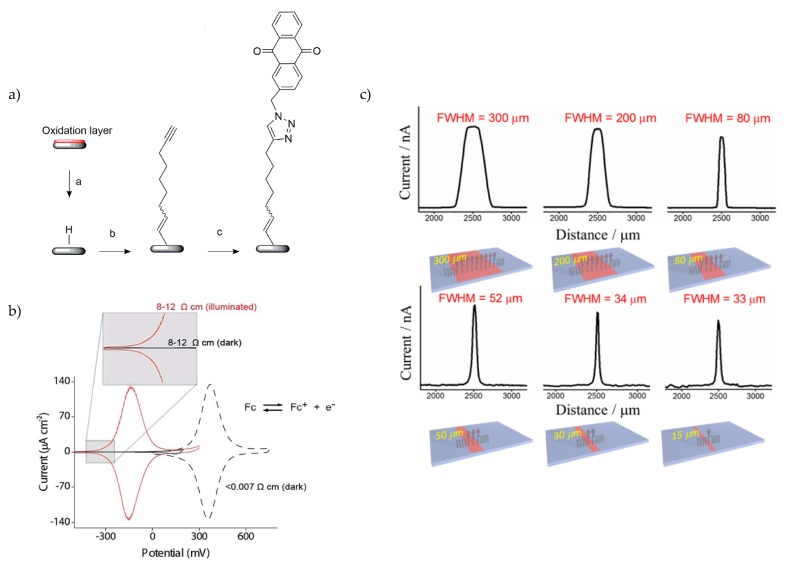
(**a**) Schematic of the process steps for addition of redox-active species (anthraquinone) on silicon. (**b**) Cycling voltammograms of highly doped p-type silicon(<0.007 Ωcm) without illumination compared to low-doped p-type silicon (1–10 Ωcm) without illumination (solid black line) and with rear-side illumination (solid red line). (**c**) Deposition of redox-active species with different widths (15–300 μm) to perform top-side laser line scans to determine the minimum spatial resolution by comparison with the full width at half maximum (FWHM) of the photocurrent. Part (a) is reprinted (adapted) with permission from Yang et al. [95] Copyright (2020) American Chemical Society. Part (b) is adapted from Reference [92] under the CC BY licence. Part (c) is reprinted (adapted) with permission from Yang et al. [96] Copyright (2020) American Chemical Society.

The different redox-active species, ferrocene (in form of azidomethylferrocene) on n-type Si and anthraquinone (in form of 2-(azidomethyl)anthracene-9,10-dione) on p-type Si, were attached to the 1,8-nonadiyne by a copper-catalyzed azide-alkyne cycloaddition reaction (CuAAc) (Figure 8a). This work demonstrates the need to use low-doped silicon since for highly doped Si (< 0.007 Ωcm); the cyclic voltammogram shows already without illumination a behaviour similiar to a metal electrode (Figure 8b, dashed black line). In contrast to low-doped Si, for cyclic voltammograms without illumination, no redox peaks are observed for low-doped n- (8–12 Ωcm) and p-doped (1–10 Ωcm, Figure 8b, solid black line) Si. In contrast, with illumination, the ferrocene-terminated n-type Si has an E_1/2_ (half-wave potential) = −140 mV *vs.* $V_{Ag/AgCl}$ and an E_1/2_ = −370 mV *vs.* $V_{Ag/AgCl}$ for the p-type Si (Figure 8b, solid red line) with anthraquinone. Additionally, alternating on- and off-switching (for 200 s each) of the charge transfer at a constant applied potential (+ 0.2 V *vs.* $V_{Ag/AgCl}$) is performed without loosing activity of the redox-species after 30 cycles [92].

The effects of the pH value of the electrolyte and light intensity on anthraquinone deposited on low-doped p-type Si (10–20 Ωcm) were further evaluated in Reference [95]. It was shown from cyclic voltammograms that E_1/2_ for modified highly p-doped Si shifts with a slope of −58.5 mV pH^−1^ and therefore behaves similar to a conventional metal electrode (anthraquinone monolayer on gold electrode; shift E_1/2_ = −59.2 mV pH^−1^), while for low-doped p-type Si, where illumination is needed for charge carrier generation, a slope of −44.0 mV pH^−1^ was obtained. As there was only a small change for the cathodic peak at different pH values, the anodic peak shifted by −83.1 mV pH^−1^. Furthermore, under a constant alkaline pH value, the anodic peak potential shows no response to an increasing light intensity, whereas the cathodic peak shifts to more positive potentials. In addition, the electron transfer rate constant for this low-doped p-type Si decreases for decreasing pH values and increases with increasing light intensities.

To evaluate the spatial resolution, anthraquinone lines of different widths (15–300 μm) were deposited on the p-doped Si. When scanning across the deposited lines, the FWHM of the related current fits to the structures down to 30 μm (Figure 8c). Furthermore, the authors stated that a top-side illumination is beneficial for the spatial resolution and additionally studied the effects of different light intensities and applied potentials [96]. Finally, as silicon as bulk material is opaque, amorphous silicon (a-Si) was deposited on ITO glass and the ferrocene was attached to 1,8-nonadiyne by the previously described CuAAc click reaction. This transparent electrode configuration is suitable for optical applications such as fluorescence imaging techniques with stimulation at the same time [97].

In conclusion, in this section, two principles for addressable photoelectrochemistry were presented. To locally change the pH value, the control of photoelectrolysis for the generation of H^+^- and OH^−^ ions has to be taken into account. Therefore, the choice of the semiconductor material, illumination time. and applied potential has to be regulated. In another approach, redox-active species ferrocene and anthraquinone were attached to the surface of the semiconductor whereby spatially resoluted Faradaic electrochemistry can be performed.

## 4. Photoelectrochemical Deposition

For certain applications, the deposition of additional materials (e.g., as co-catalyst or functional layer) on semiconductor materials is desired. Besides classical electrodeposition [98,99], also light-directed deposition can be used. Using photodeposition, no bias potential is applied, and thus, the deposition depends on reductive or oxidative photo-induced processes with a sacrifical electron donor/acceptor [100]. In contrast to this, photoelectrochemical deposition is accomplished by applying an anodic or cathodic bias potential depending on the semiconductor type. Especially, the direct patterning of these materials with advanced projection technologies makes them a serious competitor to well-established technologies such as photolitographic patterning.

For metal deposition, metal ions from the solution are reduced by photogenerated electrons in the conduction band upon illumination of the semiconductor, which is described by Equation (5):

(5) $$M^{n+}+ne_{surf}^{-}\to M_{surf}$$

where $M^{n+}$ is a metal ion of charge *n* in solution, $e^{-}$ is a photogenerated electron, and $M_{surf}$ is metal atoms on the surface.

For example, gold, copper, and nickel were deposited on p-Si and p-GaAs using standard plating solutions [101]. Photoplating currents range from 20 to 250 μA for cathodically applied potentials (−1 to −2 V *vs.* $V_{SCE}$) (SCE: saturated calomel electrode). As long as no overpotential is applied, deposition only occurs during illumination. Gold was also used on three-dimensional silicon microwires by applying −1.25 V *vs.* $V_{Ag/AgCl}$ in 0.01 M HAuCl_4_ solution [102]. Anisotropic Au deposition was demonstrated by using cylindrical microwires, whereby the gold position on the microwires mainly depends on the illumination wavelength. Furthermore, using noncylindrical microwires in combination with computer simulations (Finite Difference Time Domain Method), there was a correlation between the plating position and the concentration of generated charge carriers in the semiconductor.

For the reduction of metal ions to metals, normally p-type semiconductors are used, while for metal oxides, a n-type semiconductor is necessary [103]. Nevertheless, Au, Cu, Pt, and Ag could be deposited on n-type titanium dioxide in the presence of alcohols [104]. A TiO_2_ photoanode was biased at 0.2 V *vs.* $V_{SCE}$ in 0.1 M Na_2_SO_4_, 2 mM metal salt (H_2_PtCl_6_, HAuCl_4_, AgNO_3_, or CuCl_2_) and 10 vol% of different alcohols. As explained in Reference [104], the reaction is based on the “current doubling effect”, where holes oxidize alcohols to radicals, which inject another electron in the conduction band (mostly trap states). Furthermore, they explained that those surface electron traps can then reduce the metal ions.

**Figure 9:**
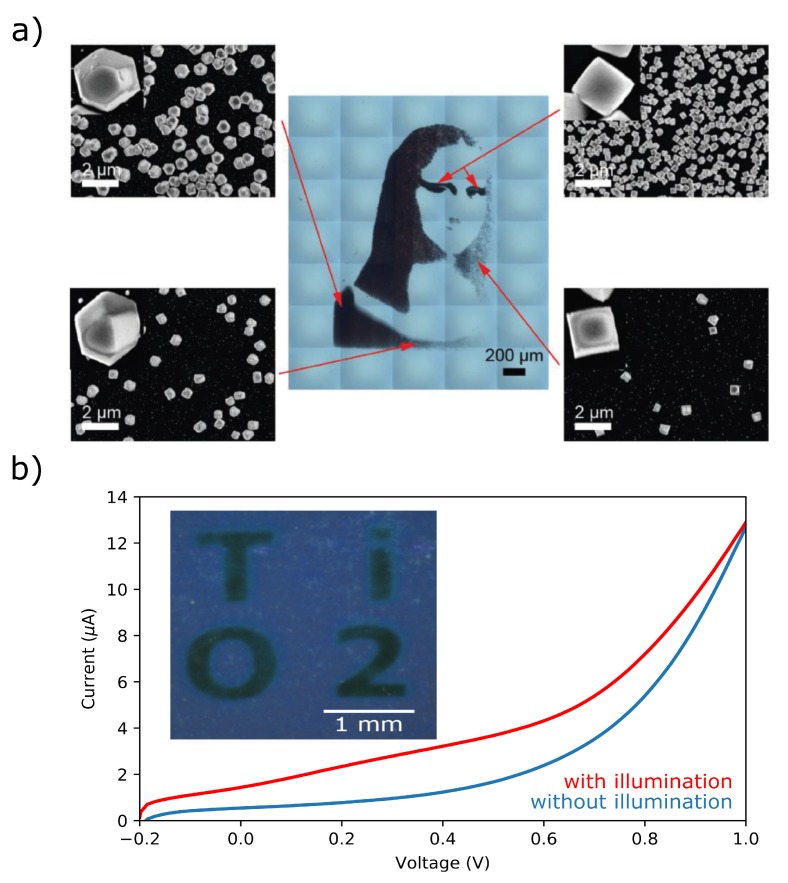
(**a**) Partial deposition of Cu_2_O by top-side illumination with a Ferroelectric Liquid Crystal on Silicon (FLCoS) display under various conditions to influence the particles’ shape and cubicity. (**b**) Snippet of the first potentiodynamic cycle with and without rear-side illumination during the polymerization of pyrrole on TiO_2_. In the inset, the deposited “TiO2” letters on the surface is shown. Part (a) is used with permission from Vogel et al. [109] Copyright (2020) Wiley.

The biggest benefit of photoelectrochemical deposition is the patterning of the desired material. One factor affecting the resolution of the structured material is the charge-carrier diffusion length within the semiconductor. Therefore, Pt, Au, and Ni–Mo were deposited on amorphous silicon, which was passivated with a thin (d = 1–2 nm) SiO_*x*_ passivation layer. As an optical system, a DMD projector with a 2× objective lens was used. For Pt (1 mM Na_2_PtCl_6_ in 0.5 M Na_2_SO_4_), a cathodic potential (−0.4 V vs. $V_{Ag/AgCl}$) was applied for 180 s. The Pt image has a minimal resolution of 100 μm. Additionally, Ni–Mo (130 mM Ni(SO_3_NH_2_), 50 mM H_3_BO_3_, and 2 mM NaMoO_4_ with pH adjusted to pH 4.0) was patterned at potentials of −0.9 V for 10 s followed by a 5 s open circuit potential (+ 0.35 V) to replenish the precursor ions [105]. Similiar to amorphous silicon, also hematite (α-Fe_2_O_3_) fullfills the requirements for a high spatial resolution through a hole diffusion length of 1–2 nm [106]. Co–Pi was deposited on hematite in different studies [106,107,108]. The oxidation of Co^2+^ to Co^3+^, triggered by a photogenerated hole was done in a 0.5 mM Co(NO_3_)_2_, 0.1 M potassium phosphate buffer at pH 7. Applying alternating voltage pulses of 0.4 V and 0 V (vs. $V_{Ag/AgCl}$) for 5 s in combination with a DMD projector leads to Co–Pi structures with radii from 20 to 200 μm, whereas for all structures, the deviation of the accomplished pattern to the illuminated area is less then 1 μm. As a last example, cuprous oxide (Cu_2_O, 50 mM CuSO_4_, 0.5 M K_2_SO_4_, and a specific amount of KCl) was deposited on amorphous silicon pasivated by a 1,8-nonadiyne layer. Pattern projection was performed by a FLCoS micromirror device. It was shown that, by changing the applied bias only, particle patterns with different cubicities can be achieved [58]. In further work, effects to change the deposited particle shape, e.g., by variation of pixel density, chloride concentration and potential were studied. As a result, by first illuminating one part of the final pattern with a defined pixel gradient followed by illuminating the second part with a different Cl^−^ concentration, areas with different Cu_2_O shapes can be implemented (Figure 9a) [109].

Besides metals, also polymers such as polystyrene [110], polyaniline [111], or polypyrrole [55,56,112,113,114,115] were sucessfully tested. Since polymerizations usually include oxidation reactions, mostly n-type semiconductors are used. In the first studies by Okano et al., polypyrrole was deposited on titanium dioxide in a three-electrode cell using a 0.1 M monomer solution of pyrrole in 0.1 M Na_2_SO_4_ with an applied potential 0.5 V *vs.* $V_{SCE}$. Masking the electrode leads to a minimum achievable line width of 45 μm [114]. It should be noted that the achievable resolution is limited by a nonsymmetrical polymerization of pyrrole [104] and that an enhanced charge transfer leads to blurring of the pattern [114]. As patterning was mostly done with shadow masks or movable lasers, it can also be spatially illuminated, e.g., by a DMD projector on a TiO_2_ LAE [116]. In a recently performed experiment by our group, it was shown that, after ten potentiodynamic cycles from −0.2 to 1.2 V *vs.* $V_{Pt}$ under illumination in 0.1 M pyrrole (0.1 M Na_2_SO_4_), the letters “TiO2” were successfully patterned on the surface (Figure 9b). Subsequently, the influence of the applied potential related to the contribution of electrochemical and photoelectrochemical polymerization [111,117] and the control of polymer homogeneity and thickness by the wavelength [115] were further analyzed. Additionally, the polymerization was performed on silicon [55], ZnO [113], and WO_3_ [111].

**Figure 10:**
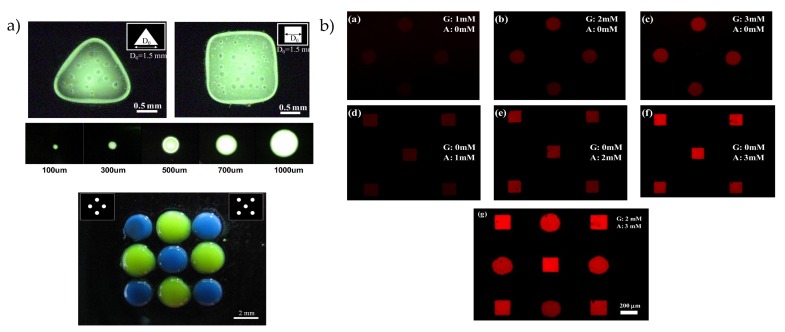
(**a**) Deposition of calcium alginate gels by rear-side illumination of hydrogenated amorphous silicon: In the top image, calcium alginate gels are deposited with different geometries and sizes. In the bottom image, the gels are deposited sequentially with two different micropatterns. (**b**) Patterning of chitosan with different geometries by rear-side illumination of an amorphous silicon photocathode: In the circular structure, glucose oxidase, peroxidase, and amplex red is added, while in the quadraric structure, alcohol oxidase is added instead of glucose oxidase. Adding ethanol or glucose increases the fluorescence intensity of the particular structure. Part (a) is used with permission from Huang et al. [85] Copyright (2020) AIP. Part (b) is adapted from Reference [87] under the CC BY licence.

The light-induced pH change introduced in Section 3 can also be used for the deposition of pH-sensitive materials. For calcium alginate gels, the decreased pH leads to a local release of calcium ions (Ca^2+^) from CaCO_3_ with a subsequent cross-linking with alginate (from sodium alginate) to form a calcium alginate gel. This gel can, for example, be used to entrap cells or enzymes [118]. On undoped amorphous silicon, calcium alginate structures with diameters down to 100 μm can be accomplished as depicted in Figure 10a [85]. It was found that the ratio between the illumination patterns and actually achieved diameter (deposition ratio) varies with illumination time, alginate and CaCO_3_ concentration, and the applied voltage. Furthermore, fibroblasts were added to the solution and, under certain conditions, the cells had a viability of more than 90%. Deposition of calcium alginate was also performed on TiOPc [86]. Different geometries were patterned, ranging from connected calcium alginate blocks and stacked layers to vessel-shaped structures by sequential illumination of the electrode.

In contrast to calcium alginate, the polysaccharide chitosan can form a hydrogel for a solution pH above pH 6.3 at illuminated areas on an amorphous silicon photocathode [87,88]. Different shapes and geometries with a minimum resolution of 40 μm were implemented. Similar to alginate, increasing the deposition time and the current density leads to an increase of the deposition ratio due to the OH^−^ ion diffusion. As shown in Figure 10b, multiple patterns were consecutively deposited with one part of the hydrogels containing glucose oxidase, peroxidase, and amplex red as a fluorescence indicator (circular shape) and the other part containing alcohol oxidase, peroxidase, and amplex red (quadratic shape). By adding glucose or ethanol, the fluorescence intensity of the particular structure increases proportianally to the concentration. Due to the different shapes, an optical readout of glucose and ethanol sensing was possible.

Upon pH changes, the light-addressed binding control of (poly)-histidine-tagged (His-Tag) proteins was also introduced [89]. A layer-by-layer composition of polystyrene sulfonate (PSS) and nickel-nitrilotriacetic (NTA) for the His-Tag binding on titanium dioxide was described. Since only the His-Tag is pH-sensitive, it can be locally adsorbed and released by pH changes triggered by illumination.

For simultaneous DNA analysis, the defined positioning of DNA microarrays on the substrate is crucial. Therefore, the spatial arrangement of DNA oligonucleotides by photoelectrophoretic transport was done with a layer structure consisting of n-type silicon (amorphous silicon), Mn_2_O_3_ passivation, and agarose-streptavidin functional layer in L-histidine solution [119]. Upon illumination, biotinylated (for streptavidin binding) and fluorescence-marked DNA oligonucleotides were bound in the illuminated areas. As for n-type silicon, the results were not reproducible; a better spatial deposition was achieved with amorphous silicon. Additionally, successfull hybridization with target DNA strands was also shown. In another approach, the electrode structure was modified and amorphous silicon was deposited on ITO glass with structured platinum pads on top. To increase the surface area, an additional porous glass layer was deposited [120]. Spatially resolved detritlyation of surface-bounded 4,4-dimethoxytrityl protecting groups by photoelectrochemically generated protons led to a subsequent hybridization of oligonucleotides.

With the previously described semiconductor electrodes, it is possible to pattern a considerable amount of materials summarized in Table 1. Depending on whether a reduction or oxidation is neccesary for deposition, a n- or p-type semiconductor has to be chosen. Furthermore, to achieve a defined pattern, the diffusion length of the semiconductor and the blurring of the deposited material with time have to be taken into account.

## 5. Conclusions

This review gives an overview on various applications for light-addressable electrodes. The basic principles of semiconductor electrochemistry were elucidated, and design strategies were explained (Section 1). For biological applications, the stimulation of neuronal cells cultivated on different semiconductor substrates was presented (Section 2). To address single cells, the material choice and design of the electrode have an influence on the spatial addressability since, depending on these factors, additional cells were stimulated outside the excited area. Area-selective photoelectrochemistry by spatially changing the analyte pH value and designing pH gradients and the possibility of light-activated electrochemistry have been depicted in Section 3. As a last topic, the possibility of structuring metals, polymers, and macromolcules with modern illumination strategies is shown. Due to the fact that the electrodes had mostly the same structure, the various applications in Section 4 can be addressed with the same setup. This opens the feasibility to combine different fields or to embed similar technologies such as the optoelectronic tweezers with the stimulation of cells. To conclude this review, with light-addressable electrodes, complex electrode structures, and a dynamic triggering of reactions such as the previously introduced cell stimulation, pH manipulation or material deposition can be achieved with a single electrode structure combined with an illumination system. This has the benefit to be easily adaptable to new applications or changes in later experimental stages in contrast to commonly used electrodes, where modifications are often followed by a redesign of the electrode.

## Figures and Tables

**Table 1:** Summary of feasible materials which can be photoelectrochemically deposited on semiconductor electrodes.

Type of Material	Deposited Material	Type of Semiconductor	References
Metal (oxide)	Au	p-Si	[101,102]
	Au	p-GaAs	[101,121]
	Ag	p-GaP	[103]
	Cu, Ni	p-Si	[101]
	Cu, Ni	p-GaAs	[101]
	Au, Cu, Pt, Ag	TiO_2_	[104]
	Pt, Au, Ni–Mo	a:Si	[105]
	Co–Pi	α-Fe_2_O_3_	[106,107,108]
	Cu_2_O	a:Si	[105]
Polymer	Polystyrene	TiO_2_	[110]
	Polyaniline	WO_3_	[111]
	Polyaniline	TiO_2_	[111]
	Polypyrrol	TiO_2_	[56,111,112,114,115,122]
	Polypyrrol	n-Si	[55,92]
	Polypyrrol	ZnO	[113]
Others	Calcium alginate gels	a:Si	[110]
	Calcium alginate gels	TiOPc	[85,86]
	Chitosan	a:Si	[87,88]
	Polyhistidine-tagged proteins	TiO_2_	[89]
	DNA oligonucleotids	n-Si	[119]
	DNA oligonucleotids	a:Si	[120]
